# Contrasting Roles for CD4 vs. CD8 T-Cells in a Murine Model of Virally Induced “T1 Black Hole” Formation

**DOI:** 10.1371/journal.pone.0031459

**Published:** 2012-02-13

**Authors:** Istvan Pirko, Yi Chen, Anne K. Lohrey, Jeremiah McDole, Jeffrey D. Gamez, Kathleen S. Allen, Kevin D. Pavelko, Diana M. Lindquist, R. Scott Dunn, Slobodan I. Macura, Aaron J. Johnson

**Affiliations:** 1 Department of Neurology, Mayo Clinic, Rochester, Minnesota, United States of America; 2 Department of Neurology, University of Cincinnati, Cincinnati, Ohio, United States of America; 3 Department of Pathology and Immunology, Washington University in St. Louis, St. Louis, Missouri, United States of America; 4 Department of Immunology, Mayo Clinic, Rochester, Minnesota, United States of America; 5 Imaging Research Center, Cincinnati Children's Hospital Medical Center, Cincinnati, Ohio, United States of America; 6 Department of Biochemistry, NMR Core Facility, Mayo Clinic, Rochester, Minnesota, United States of America; Washington University, United States of America

## Abstract

MRI is sensitive to tissue pathology in multiple sclerosis (MS); however, most lesional MRI findings have limited correlation with disability. Chronic T1 hypointense lesions or “T1 black holes” (T1BH), observed in a subset of MS patients and thought to represent axonal damage, show moderate to strong correlation with disability. The pathogenesis of T1BH remains unclear. We previously reported the first and as of yet only model of T1BH formation in the Theiler's murine encephalitis virus induced model of acute CNS neuroinflammation induced injury, where CD8 T-cells are critical mediators of axonal damage and related T1BH formation. The purpose of this study was to further analyze the role of CD8 and CD4 T-cells through adoptive transfer experiments and to determine if the relevant CD8 T-cells are classic epitope specific lymphocytes or different subsets. C57BL/6 mice were used as donors and RAG-1 deficient mice as hosts in our adoptive transfer experiments. In vivo 3-dimensional MRI images were acquired using a 7 Tesla small animal MRI system. For image analysis, we used semi-automated methods in Analyze 9.1; transfer efficiency was monitored using FACS of brain infiltrating lymphocytes. Using a peptide depletion method, we demonstrated that the majority of CD8 T-cells are classic epitope specific cytotoxic cells. CD8 T-cell transfer successfully restored the immune system's capability to mediate T1BH formation in animals that lack adaptive immune system, whereas CD4 T-cell transfer results in an attenuated phenotype with significantly less T1BH formation. These findings demonstrate contrasting roles for these cell types, with additional evidence for a direct pathogenic role of CD8 T-cells in our model of T1 black hole formation.

## Introduction

Multiple sclerosis is the leading cause of disability among young adults in the western world [1]. MRI findings in MS represent important components of the diagnostic criteria and are extensively used in clinical disease monitoring [2]. In general, T2 hyperintense lesion-based MRI features in MS ,including lesion load or number of lesions, show only a limited correlation with neurological outcome and future disability risk; however, the presence of chronic lesional T1 hypointensity or T1 black holes (T1BH) is associated with a more disabling course [3], [4]. T1BH is thought to represent axonal damage [5], [6]. The exact pathomechanism leading to the formation of T1BH remains poorly understood due to the lack of appropriate experimental model systems and inability to mechanistically address this issue in clinical research.

TMEV infection in mice is an accepted model of multiple sclerosis [7], [8]. In this model, mice of susceptible genetic background develop a biphasic disease; the late or chronic phase is characterized by chronic-progressive demyelination. In contrast to the most commonly used experimental allergic encephalomyelitis (EAE) models, the initial target of the immune response in the TMEV models is the viral infection itself; however, in later stages of the disease, the immune response also includes myelin self-epitopes via epitope spread [9]. C57BL/6 mice, infection with Theiler's Murine Encephalomyelitis Virus (TMEV) results in the MRI phenomenon of T1BH formation [10]. It is important to note that C57BL/6 mice represent a resistant strain, meaning that chronic-progressive demyelination doesn't develop in this model. The observed T1 black holes appear in the context of acute neuroinflammation in the early stage of viral infection; however, axonal damage, the most specific and pathognomonic process that characterizes T1 black holes in MS, is also observed in these areas in our model [10], [11]. Similarly to the original observations in MS, tissue studies in our model demonstrate extensive axonal and neuronal damage in these areas [12]. As demonstrated by our team earlier, CD8 T- cells are the main contributors in the pathogenesis of T1 hypointense lesions in this model [10]. CD8 T-cells have also been associated with axonal injury in the TMEV model [13], [14], [15], [16], [17], contribute to the development of autoimmune responses in this model [18] and also play a critical role in inducing severe blood-brain barrier permeability under specific conditions [19], [20], [21], [22].

Traditionally, CD4 T cells, including Th1 and Th17 cells are considered the most significant mediators in the pathogenesis of MS and its key animal model, EAE (experimental allergic encephalomyelitis) [23]. While subsets of CD 4 T cells are common targets in therapeutic efforts directed at MS, recent pathology reports also indicate an important role for CD8 T cells. CD8 T cells are the most numerous lymphocyte subpopulations in MS lesions and in normal appearing MS tissue, regardless of MS subtype or stage of lesion formation [15], [24]. CD8 T-cells were also observed in close proximity to damaged neurons in axons both in MS and our model [15], [25]. Despite these observations, the role of CD8 T-cells remains controversial, as some studies suggest that CD8 T-cells are mainly regulators and suppressors of the inflammatory activity. [15], [24] Therefore, the contribution of CD8 T cells in MS pathogenesis in the form of axonal disruption and new lesion formation needs to be further defined.

Our current study had *two main goals:* (1) to establish whether the pathogenic CD8 T-cells in our model are classic viral epitope specific cytotoxic lymphocytes or other CD8+ immune cell types, and (2) to investigate the potential role of CD4 T cells in the process of T1BH formation.

In our experiments, we utilized transgenic mice with no adaptive immune system (RAG-1^−/−^ mice). We adoptively transferred CD8+ or CD4+ cells from donor C57BL/6 mice into RAG-1^−/−^ mice [25]. These experiments were designed to demonstrate if the immune system's capability to generate T1BH can be restored by the above cell transfers. In a separate experiment, we utilized a peptide depletion technology to selectively eliminate CD8 T-cells recognizing the immunodominant peptide in our model [26]. We know from previous experiments in this model that over 70% of brain infiltrating CD8 T-cells recognize a specific viral capsid peptide, VP2_121–130_ [26], [27], [28]. By intravenously injecting this peptide one day prior to disease induction with TMEV, we can effectively eliminate CD8 T-cells that recognize this peptide [26], [27].

Our experiments demonstrate a surprising contrast in the contribution by CD8 vs. CD4 T-cells in T1BH formation, and confirmed that T1BH formation is driven mainly by epitope specific CD8 T-cells in our model.

## Materials and Methods

### Mice

C57BL/6J and RAG-1 deficient mice on C57BL/6 background were purchased from the Jackson Laboratory in Bar Harbor, ME. The number of mice used is as detailed below. The experiments were approved by the local Institutional Animal Care and Use Committee at the University of Cincinnati (06-10-09-01) and at Mayo Clinic, Rochester, Minnesota (A29509). TMEV infection using the Daniels strain was induced via intracerebral inoculation of 200,000 PFU using standard methods described earlier [29], [30], [31].

### Peptide depletion experiment

In the experiments determining whether VP2 epitope specific CD8 T-cells contribute to the process of T1BH formation, we used epitope depletion by intravenous administration of VP2_121–130_ peptide one day prior to TMEV infection, as described in details earlier [27]. In this study, 8 TMEV infected mice underwent VP2 depletion, and 8 TMEV infected mice received irrelevant E7 peptide injection as negative controls. The E7 peptide is derived from the human papilloma virus, which has no relevance to TMEV infection [27].

### Isolation of Brain Lymphocytes

As published by our team earlier [12], whole brains were strained through a nylon mesh 100-µm filter into RPMI (MediaTech Inc., Herndon, VA), and 700 µg of collagenase type 4 (Worthington, Lakewood, NJ) was added to each 5-ml tissue suspension. Suspensions were incubated in a water bath at 42°C for 45 minutes. Each 5-ml suspension was then added to 50 ml high speed centrifuge tubes (Nalge Nunc International, Rochester, NY) containing a solution of 1 ml of 10× PBS, 9 ml of Percoll (Sigma-Aldrich GmbH, Steinheim, Germany), and 35 ml of RPMI. Cell suspensions were then spun at 10,000 rpm (Sorvall SS-34 rotor) for 30 minutes (× g max = 11962.6). The lymphocyte layer present in the bottom 5 ml of solution was collected. This lymphocyte layer was then suspended into 50 ml conical tubes (Becton Dickinson, Franklin Lakes, NJ) and diluted with RPMI to a total volume of 50 ml. Cell suspensions were then spun at 300× g for 10 minutes on a Sorvall Legend RT tabletop centrifuge. Media was aspirated off, and cell pellets were resuspended in RPMI media.

### FACS analysis

Isolated brain lymphocytes were resuspended in FACS buffer (1× PBS, 1% FCS and 0.025% sodium azide) and incubated with Db:VP2^121–130^ tetramer or Db:E7 tetramer for 40 minutes followed by a 20 minute incubation with anti-CD4 and anti-CD8. Cells were then rinsed twice with FACS buffer and fixed in 1% PFA in 1× PBS. Samples were run on a BD LSR II flow cytometer (BD Biosciences, San Jose CA) and analyzed using FACS Diva software (BD Biosciences, San Jose CA).

### Studies of viral load

We performed plaque assays to determine viral load using established methods [32], [33]. Briefly, 7-day TMEV infected brains were collected in Dulbecco's medium and frozen at −80°C. We studied 3 brains per group. The brains were then thawed, homogenized, and sonicated using two 90 second pulses. This solution was then centrifuged at 2000 rpm for 10 minutes and the supernatant was removed.

The plaque assays were performed using 10-fold dilutions of brain homogenate. The serial dilutions aliquots were inoculated onto susceptible L2 cell monolayers. Each dilution is plated in duplicate to enhance accuracy. L2 cells were plated at a density of 1×10^5^/well in 12-well plates the day before. The cells were incubated at 37°C for 1 h before the addition of 1 ml of DMEM containing 2% FBS and 0.5% low-melting-point agarose. When the plates are incubated, the original infected cells release viral progeny. Viral spread is restricted to neighboring cells by the gel. Consequently, each infectious particle produces a circular zone of infected cells called a plaque. Eventually the plaque becomes large enough to be visible to the naked eye. After 72 h at 37°C, the cells were fixed and stained with cresyl violet to enhance the contrast between the living cells and the plaques.

Viral titers were calculated in plaque-forming units (PFU) per milliliter. To determine the virus titer, the plaques are counted at the lowest dilution that forms plaques in the plate wells. To calculate the titer of virus the plagues from the lowest dilution to form plaques were counted. The duplicates for each dilution were counted and averaged. The average was then multiplied by 50 and the dilution factor that was counted. The resulting number is the PFU per well.

### Adoptive transfer experiments

To determine the role of CD8 and CD4 T cells in general, we utilized mice that lack an adaptive immune system, and as such, they lack CD 4 and CD 8 T cells. These RAG-1^−/−^ mice otherwise have the same genetic background as C57BL/6 mice. We harvested CD8 and CD 4 T cells from spleens of C57BL/6 mice, and transferred these into irradiated RAG-1 −/− recipient mice as described earlier [25]. Briefly, spleens of GFP+ mice (C57BL/6-Tg(UBC-GFP)30Scha/J mice, # 004353, The Jackson Laboratory, Bar Harbor, ME) were removed and strained through a nylon mesh 100-µm filter. CD8+ and CD4+ cells were purified from the resulting lymphocyte population using MACS LS cell purification columns (Miltenyi Biotec, Auburn, CA) according to the manufacturer's protocol, resulting in 95% purity as determined by flow cytometric analysis (data not shown). C57BL/6 mice were irradiated with 400 rads of γ-radiation then received 10^6^ CD8+ or CD4+ positively sorted spleen cells via tail-vein injection. Positive sorting does not affect functions of CD4 or CD8 T cells.

### MRI acquisition and analysis

In each case, the formation of T1BH was monitored by volumetric MRI: a volume acquisition T1BH-s were defined as focal cerebral hypointensity as demonstrated by our team earlier [10]. T1 weighted spin echo sequence was used, with 200 µm isometric resolution using a Bruker Biospec 7 Tesla horizontal bore small animal MRI system, on day 7 following disease induction with TMEV, as described earlier [10], [34]. We utilize standard spin echo sequences to conform with the original study demonstrating T1BH in MS [3]. For volumetric analysis, the 3D ROI tool was used in Analyze 9.1 (Mayo Clinic, Biomedical Imaging Resource) [35], [36]. Since the analysis is semi-automated, we needed to standardize the analysis methods and use 2 investigators to analyze each dataset at least twice. Their intra-and inter-rater reliability was superior, as reported earlier [37]. The outcome analyzed in our studies was the total volume of T1BH per animal.

## Results

### Adoptive cell transfer experiments

**Figure 1:**
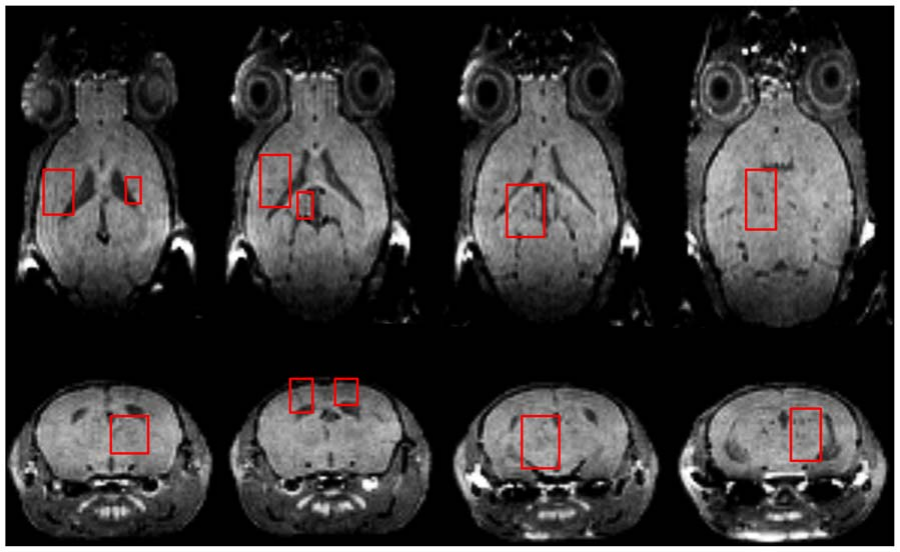
Example for T1 black hole formation in 7-day TMEV infected C57BL/6J mice. Top row: axial images, bottom row: coronal images extracted from a representative mouse. To provide guidance, some (but not all) areas containing T1 black holes are indicated with red frames. These are located in diverse areas of the brain, but mostly in periventricular areas, in the thalamus, hippocampus, white matter, corpus callosum and even in the cortex.

**Figure 2:**
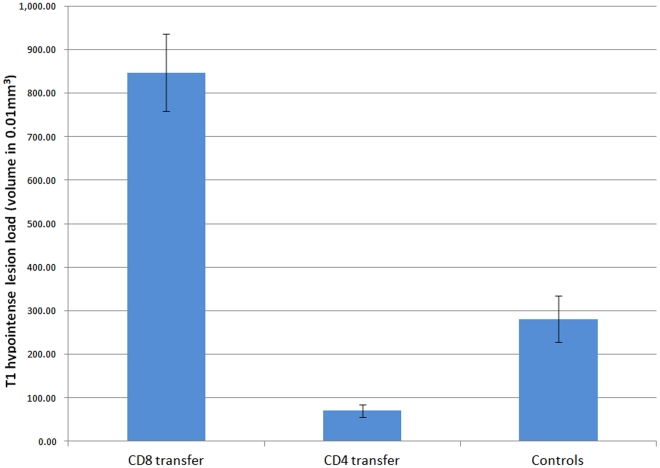
MRI T1BH volumetry results in adoptive transfer experiments using RAG 1 −/− recipient mice. The bar chart shows the mean T1BH volumes in CD8 and CD4 adoptive transfer experiments. Controls represent sham transfer (PBS injection) following irradiation and infection. CD8 T cell transfer resulted in 3-fold increase of T1BH formation, while CD4 transfer resulted in a 2-fold decrease. Error bars represent SD.

T1BH-s were observed in all TMEV infected experimental groups (Figure 1). The cell transfer experiments revealed that CD8 T cell recipient mice developed an average T1BH volume over 3 fold higher compared to sham transfer controls (p = 0.0002) (Figure 2). The detected total T1BH volumes per animal were comparable to our original findings in C57B6/J mice [10]. In sharp contrast, our CD4 transfer experiments demonstrated a surprising finding: CD4 T cells actively inhibited T1BH formation in our model. An almost 4-fold reduction was detected (p = 0.0006) in T1BH volume compared to sham treated irradiated infected controls. The observed low-level T1BH formation among recipients of sham transfer is thought to be mediated either by innate immune cells, most likely neutrophils, microglia or macrophages; or via direct viral cytopathic effects. These alternative mechanisms are also the subject of ongoing investigations in our laboratory. We did not perform CD4 or CD8 transfers to uninfected hosts, as homing of immune cells in that condition is not expected.

### FACS analysis of brain infiltrating lymphocytes

**Figure 3:**
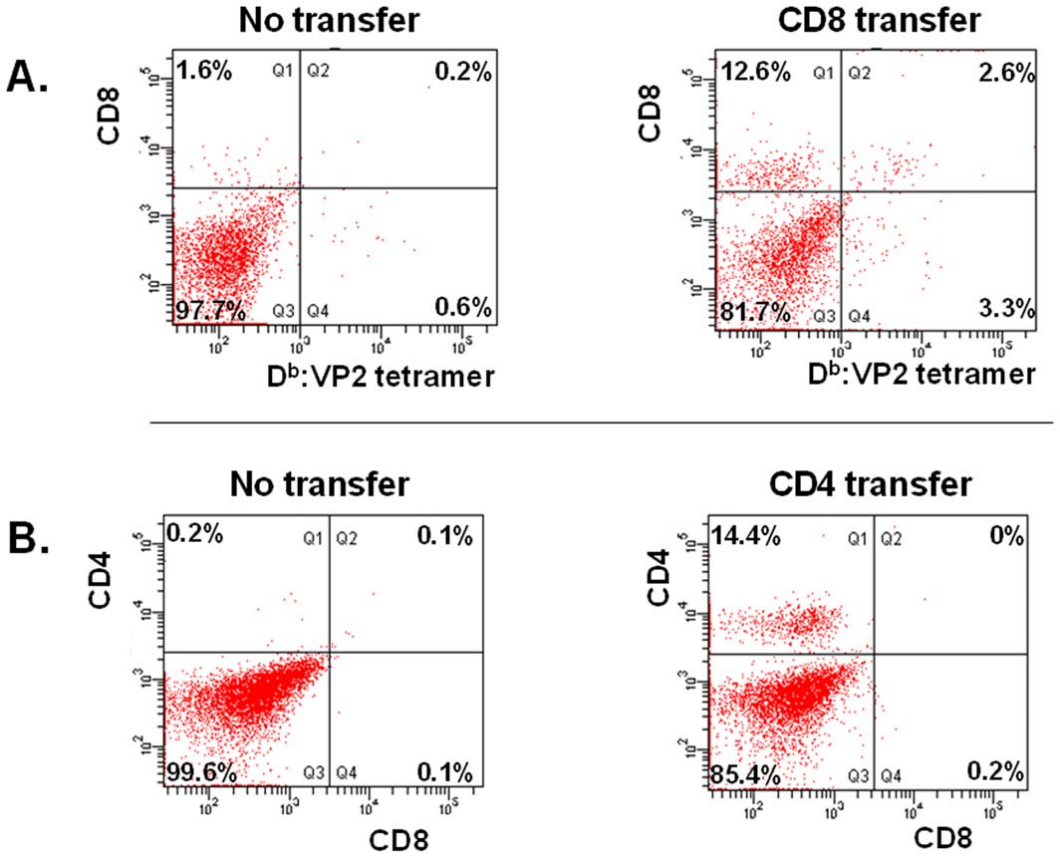
FACS analysis of brain infiltrating immune cells on day 7 following TMEV infection. Figure 3A (top half) shows transfer efficiency following adoptive transfer of CD8+ splenocytes from C57BL/6 donors into RAG-1 deficient hosts. Note that with sham transfer, only 1.6% of BIL-s is CD8+ and only 0.2% recognizes VP2 epitopes, whereas transfer of CD8+ cells results in 12.6% CD8+ positivity and 2.6% positivity for VP2 peptide. The 3.3% VP2+ CD8− cells are overall unidentified, and potentially represent dead cells that bind tetramer, auto fluorescing CNS cells, dying CD8+ tetramer+ cells that have become CD8− tetramer+, or NK cells with receptors specific for class I. Figure 3B (lower half) describes transfer efficiency in our CD4 transfer experiments. With sham transfer, only 0.2% of BIL-s is CD4+, whereas with CD4 transfer, 14.4% positivity is demonstrated.

Following the MRI acquisition experiments, the animals were sacrificed on the same day and their brains were extracted for flow cytometric analysis of brain infiltrating lymphocyte subsets. (Figure 3.) We were specifically interested in transfer efficacy, and in the proportion of CD8 T-cells recognizing VP2 epitopes. In brains with sham transfer, 1.6% of cells were CD8+ positive vs. in brains with CD8 splenocyte transfer, were 12.6% positivity was demonstrated (Figure 2A). Using tetramer technology, we demonstrated that a total of 2.6% of CD8+ cells were also VP2 121–130 specific.

CD4 transfer was also monitored by FACS (Figure 3B). 14.4% of brain infiltrating immune cells were CD4+ and CD8− following CD4+ splenocyte transfer; whereas only 0.2% were CD4+ CD8− after sham transfer.

### Viral load analysis

**Figure 4:**
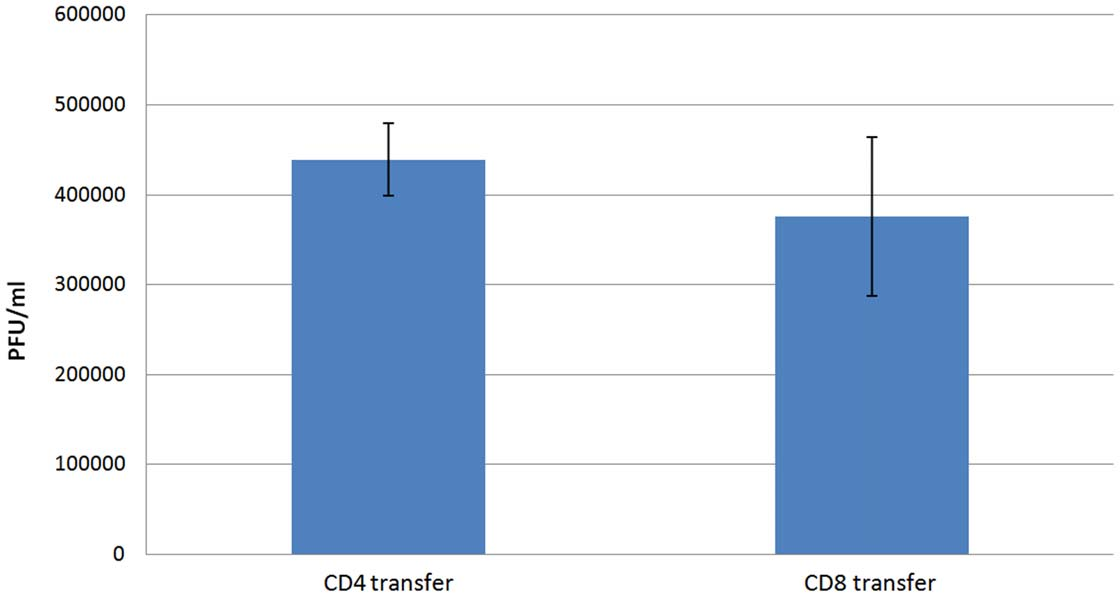
Differences in viral load. We measured TMEV viral load on day 7 following TMEV infection of RAG-1 deficient mice that underwent adoptive transfer of CD4 or CD8 T-cells, respectively. Overall, the viral loads aren't different (p = 0.62) between the two groups, demonstrating that the observed T1BH formation isn't directly related to viral load.

To determine whether differences in viral load may explain the observed differences in T1BH volume between our CD8 and CD4 adoptive transfer groups, we performed plaque assays. These assays revealed that there was no significant difference (p = 0.62) in viral load between CD8 and CD4 adoptive transferred RAG-1 deficient mice on day 7 of TMEV infection (Figure 4).

### Epitope specific CD8 T-cell depletion experiments

**Figure 5:**
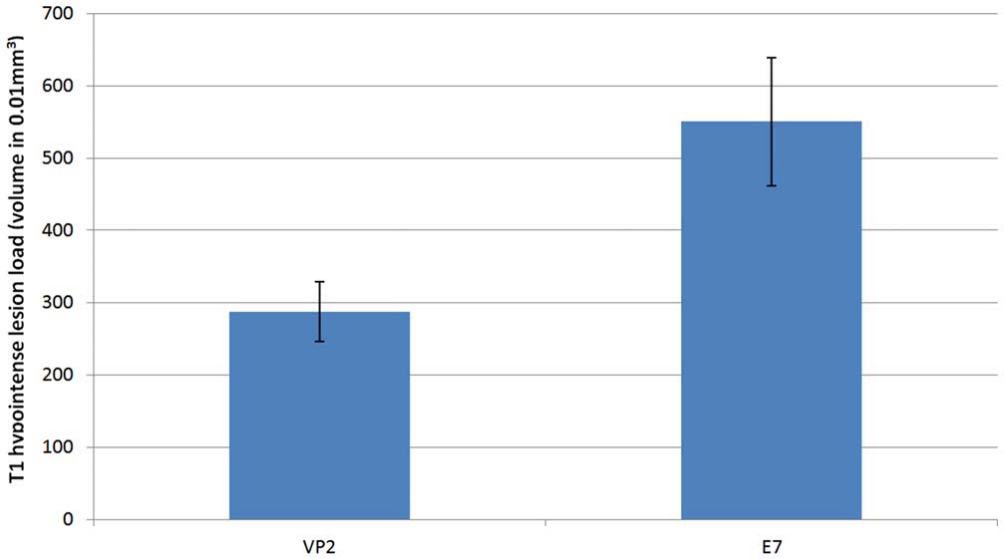
MRI T1BH volumetry results following peptide depletion. VP2 is the viral epitope recognized by over 75% of all CNS infiltrating CD8 T cells in the context of TMEV infection of C57BL6/J mice. E7 is an irrelevant viral petide. Note the significant reduction in T1BH formation by peptide-induced depletion (injection of VP2 peptide 1 day prior to infection). Y axis: volume units in 0.01 mm^3^. Error bars represent SD.

Depleting the CD8 T cells that recognize the VP2 immunodominant peptide (Figure 5) resulted in a significant 2-fold reduction of T1BH formation compared to sham treatment with E7 peptide (p = 0.017). This suggests that the vast majority of CD8 T-cells contributing to T1BH formation are classic epitope specific cytotoxic lymphocytes.

## Discussion

The above results reinforce that CD8 T cells play the predominant role in the pathogenesis of T1BH formation, and clarifies that the leading contribution in this process is by epitope specific cytotoxic CD8 T cells. Since a small degree of T1BH formation is observed even in control animals (infected RAG-1 −/− mice receiving sham transfer) it is likely that either direct cytopathic effects or cells of the innate immune system, likely neutrophils, also contribute to this process. Meanwhile, when CD4 cells are transferred to animals with deficient adaptive immunity, they appear to play a preventive role in T1BH formation and significantly reduce the observed baseline T1BH formation.

T1BH-s are thought to represent axonal and/or neuronal damage in MS [6], [38], [39]. In the TMEV model, viral replication mostly takes place in neurons in the stage when our experiments were conducted [40], [41], and at this stage and in this strain, demyelination is not observed, unless stimulated by the intravenous injection of the immunodominant VP2 peptide, as demonstrated by our team earlier [19], [42]. We therefore hypothesize that cytotoxic CD8 T cells targeting the immunodominant VP2 epitope are targeting neurons and axons, consistent with our recent published studies [12]. By transferring CD8 T-cells into CD8 deficient infected hosts, we were able to restore T1BH formation. In addition epitope specific elimination of VP2 specific CD8 T-cells resulted in reduced T1BH formation. Altogether, these observations very strongly suggest that T1BH formation in this model is related to neuronal and axonal injury caused directly or indirectly by CD8 T-cells. Given that CD8 T-cells are the most numerous lymphocytes in MS lesions regardless of stage of lesion formation, and given that CD8 T-cells have been observed strongly apposed to neurons and axons in MS tissue, we propose that a similar CD8 T cell driven mechanism as observed in our model may be responsible for T1BH formation in MS [43], [44]. Another key analogy between our model and MS-related T1BH formation is that in both cases, axonal injury appears to be the main tissue damage captured by this MRI finding, which is further underlined by the fact that demyelination is absent at this stage and in this strain in our model. [3], [5], [45]

The surprising protective role of CD4 T cells may be explained by previously published findings related to CD4 T-cell biology in TMEV infection. CD4 T-cells can play both protective and pathogenic roles in TMEV infection. During the acute neuroinflammatory stage of TMEV infection, CD4+ Th1 T-cells appear to control the viral infection [46], [47], although we did not notice significant differences in viral load in our experiment in day 7; therefore the observed protective role is unlikely to be related to this effect. Furthermore, experimental neutralization of a classic CD4 T-cell cytokine, IFN-γ, resulted in significantly accelerated disease onset [48]. IFN-gamma was also demonstrated to play a critical role in protecting spinal cord neurons from persistent TMEV infection and death [49]. On the other hand, these cells also play a pathogenic role in the chronic stages of TMEV infection of susceptible mice, where neutralization of IL-12, a classic CD4 Th1 cytokine, resulted in an attenuated disease phenotype with decreased demyelination [50]. CD4 T-cells with a Th2 phenotype have been proposed to overall suppress inflammation and demyelination: IL-4 treatment during the early chronic phase of TMEV infection resulted in a more benign disease phenotype, with reduced anti-TMEV antibody responses [51]. However, this observation is not applicable to our experimental system for two reasons: one, the utilized RAG-1 deficient mice are unable to mount an antibody response; two, we are studying the acute neuroinflammatory stage of TMEV infection. Overall, in the classic TMEV induced biphasic MS models, it remains unclear whether Th2 cytokines play a protective (suppression of pathogenic CD4 Th1 cells) or pathogenic role (by increasing antibody production) in demyelinating disease [52].

A recent novel observation suggests that Th17 cells, a subset of CD4 T cells prolong neuronal survival in the TMEV model in vitro [53]. Such neuroprotective effect may also have contributed to the observed attenuated MRI phenotype in our study. In addition, Th17 mediated suppression of CTL function was also observed in TMEV infection, which may also theoretically lead to reduced T1BH formation, but not in our model system, as endogenous CD8 T-cells are not present in our host mice.

In the TMEV model, BALB/cByJ mice are resistent while BALB/cAnNCr mice are susceptible to the development of chronic demyelination. The susceptibility of BALB/cAnNCr mice is thought to be directly caused by a defective/absent CD4+ T-cell subset, providing another example how CD4+ T-cells may regulate the overall phenotype observed in this model, acting in the earliest, most acute stage immediately after infection [54].

We constantly observe low level but clearly detectable T1BH formation in RAG-1 deficient mice (Figure 2) [10]. We therefore suspect that cells of innate immunity, likely neutrophils, macrophages or microglia may contribute to axonal/neuronal damage and resultant T1BH formation, which is an active area of research in our laboratory. We also hypothesize that the presence of CD4 T-cells in our adaptive transfer model may result in the attenuation of this innate immune response leading to the observed reduction in T1BH formation. The exact mechanism for the above is to be determined in ongoing and future experiments in our laboratory.

In conclusion, these findings demonstrate a contrasting role for CD4 vs. CD8 cells in T1BH formation in the TMEV model of MS. Further investigations in our model will clarify the role and significance of CD8 T-cells in axonal and neuronal damage, and the role of CD4 T-cells in the observed protective process.
